# Risk factors for hospital-acquired influenza A and patient characteristics: a matched case-control study

**DOI:** 10.1186/s12879-020-05580-9

**Published:** 2020-11-19

**Authors:** Kui Yang, Ni Zhang, Chunchen Gao, Hongyan Qin, Anhui Wang, Liqiang Song

**Affiliations:** 1 Department of Pulmonary and Critical Care Medicine, Xijing Hospital, Fourth Military Medical University, No.127, Changle West Road, Xincheng District, Xi’an, 710032 Shaanxi Province China; 2 Department of Basic Medicine, Xi’an Medical University, No.1, Xin-Wang Road, Weiyang District, Xi’an, 710021 Shaanxi Province China; 3 Department of Medical Genetics and Developmental Biology, Fourth Military Medical University, No.169, Changle West Road, Xincheng District, Xi’an, 710032 Shaanxi Province China; 4 Department of Epidemiology, School of Preventive Medicine, Fourth Military Medical University, No.169, Changle West Road, Xincheng District, Xi’an, 710032 Shaanxi Province China

**Keywords:** Influenza, human, Nosocomial infection, Hospital-acquired influenza a, Risk factors

## Abstract

**Background:**

While hospital-acquired influenza A results in an additional cost burden and considerable mortality in patients, its risk factors are unknown. We aimed to describe the characteristics of patients vulnerable to hospital-acquired influenza A and to identify its risk factors to assist clinicians control hospital-acquired infections and reduce the burden of treatment.

**Methods:**

A case-control study was conducted among hospitalized patients aged ≥18 years at a tertiary level teaching hospital during the 2018–2019 influenza A season. Patient data were retrieved from hospital-based electronic medical records. Hospital-acquired influenza A was defined as a case of influenza A diagnosed 7 days or more after admission, in a patient with no evidence of influenza A infection on admission. The controls without influenza A were selected among patients exposed to the same setting and time period. We identified risk factors using conditional logistic regression and described the characteristics of hospital-acquired influenza A by comparing the clinical data of infected patients and the controls.

**Results:**

Of the 412 hospitalized patients with influenza A from all the departments in the study hospital, 93 (22.6%) cases were classified as hospital-acquired. The most common comorbidities of the 93 cases were hypertension (41.9%), coronary heart disease (21.5%), and cerebrovascular disease (20.4%). Before the onset of hospital-acquired influenza A, patients presented more lymphocytopenia (51.6% vs 35.5%, *P* = 0.027), hypoalbuminemia (78.5% vs 57.0%, *P* = 0.002), and pleural effusion (26.9% vs 9.7%, *P* = 0.002) than the matched controls. Infected patients also had longer hospital stays (18 days vs 14 days, *P* = 0.002), and higher mortality rates (10.8% vs 2.2%, *P* = 0.017) than the matched controls. Lymphocytopenia (odds ratio [OR]: 3.11; 95% confidence interval [CI]: 1.24–7.80; *P* = 0.016), hypoalbuminemia (OR: 2.24; 95% CI: 1.10–4.57; *P* = 0.027), and pleural effusion (OR: 3.09; 95% CI: 1.26–7.58; *P* = 0.014) were independently associated with hospital-acquired influenza A.

**Conclusions:**

Lymphocytopenia, hypoalbuminemia and pleural effusion are independent risk factors that can help identify patients at high risk of hospital-acquired influenza A, which can extend hospital stay and is associated with a high mortality.

**Supplementary Information:**

The online version contains supplementary material available at 10.1186/s12879-020-05580-9.

## Background

The mutation rate of influenza A virus is the highest among the three reported subtypes of human influenza virus (A, B, and C) [1]. Large-scale human-to-human transmission of influenza A virus can occur biannually, in winter and spring. Outbreaks of influenza A virus infection in hospitalized patients have been reported in a variety of clinical settings including neonatal intensive care units [2], geriatric wards [3], and hematology units [4]. Hospital-acquired influenza A attacks hospitalized patients with a primary disease and results in additional treatment burdens and adverse health consequences.

Hospital-acquired influenza A may be associated with a poor prognosis. In Germany, a case fatality rate of 9% was mainly associated with influenza virus A (H1N1) pdm09 [5], and in Sweden, a hospital-acquired influenza A with a case fatality rate of 9.6% has been reported [6]. Moreover, hospital-acquired influenza A has been reported to be an independent factor associated with mortality among patients admitted to an intensive care unit (ICU) [7].

Hospitals are semi-closed settings and hospitalized patients are in close contact with each other. Patients with influenza A in the incubation period can be asymptomatic sources of infection [8]. The incubation period may be as long as 7 days [9], which makes the prevention and control of influenza A among hospitalized patients a major challenge. Therefore, the early recognition of patients with a high risk of hospital-acquired influenza A can play an important role in the prevention of influenza A outbreaks among hospitalized patients.

Although the clinical and epidemiological features of hospital-acquired influenza A are well-documented [10, 11], there have been few studies on its risk factors. While other published studies chose community-acquired influenza A cases as controls [7, 12], our study selected controls that were hospitalized in the same department and during the same time period without acquiring the infection, and thus matching cases and controls more reliably.

This study aimed to identify the risk factors for hospital-acquired influenza A, so that vulnerable individuals could be identified at an early stage.

## Methods

### Design and study population

This single-center retrospective matched case-control study analyzed the medical records of patients with hospital-acquired influenza A and matched controls from a tertiary level teaching hospital in Xi’an China during the 2018–2019 influenza A season from December 1, 2018 to April 31, 2019.

**Fig. 1:**
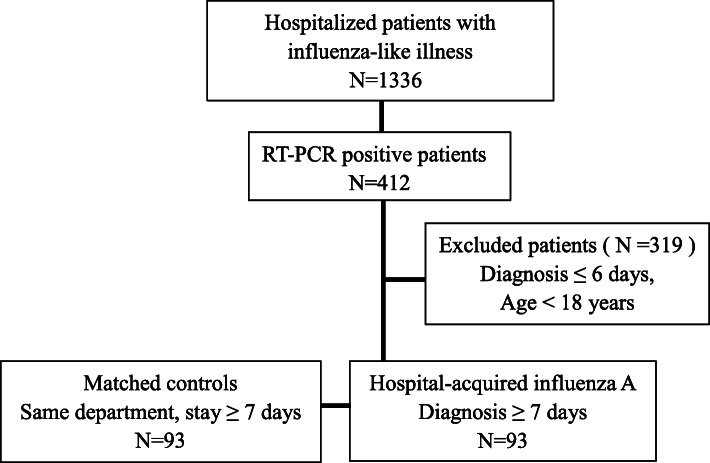
A flow chart showing the patient selection

Patients hospitalized for more than 24 h with an influenza-like illness (ILI), and confirmed as having influenza A by a laboratory test conducted on the day that they developed the ILI, were selected from the wards of different departments. The preliminary objective was to describe the epidemiological characteristics of influenza A diagnosed among hospitalized patients. Subsequently, we classified the cases diagnosed 7 days or more after admission as hospital-acquired influenza A (Fig. 1).

The controls were patients without influenza A and were matched in a 1:1 ratio according to hospital department, age, and period of hospitalization (Fig. 1). Patients less than 18 years old were excluded from this study. Formatted case report forms (CRFs) were used to collect clinical information of hospital-acquired influenza A patients and the matched controls.

### Case definitions

According to the 2018 version of the Diagnostic and Treatment Protocol for Influenza [8] released by the National Health and Family Planning Commission of the People’s Republic of China, the main manifestations of ILI were fever, headache, cough, nasal congestion, runny nose, and systemic symptoms such as muscle and joint aches, fatigue, and loss of appetite. During the influenza season, patients who met these ILI criteria were considered to be suspected influenza A cases. A confirmed diagnosis of influenza A was based on the manifestation of ILI symptoms combined with a positive result from a real-time reverse transcription polymerase chain reaction (RT-PCR) test for influenza A performed using a nasopharyngeal swab sample. Considering that the maximum incubation period for influenza A was up to 7 days [9], hospital-acquired influenza A was defined as cases diagnosed 7 or more days after admission, among patients who had no evidence of influenza A infection on admission. In addition, pneumonia and chronic obstructive pulmonary disease patients with respiratory symptoms who screened negative for influenza A virus upon admission and subsequently tested positive 7 or more days later, were eligible for inclusion.

Since the influenza A virus is spread mainly through aerosols, droplets, or by contact [13], with age being a potential confounding factor for the influenza A virus infection [14], we matched every hospital-acquired influenza A patient with a clearly defined control. The control was required to be exposed to the same setting for the same period of time, in other words, the control was required to have been hospitalized for 7 days or longer in a ward of same department on the date when the matched patient was diagnosed with influenza A, and the age difference was required to be within 5 years.

There were two types of controls: The first type consisted of patients who showed no ILI manifestation during hospitalization, with no need for a RT-PCR test; with the second type being patients who showed respiratory symptoms similar to ILI during hospitalization but who tested negative for influenza A virus; their respiratory symptoms clearly caused by bacterial pneumonia or chronic obstructive pulmonary disease.

### Data collection

A CRF was designed for data collection, including demographics (age, sex), date of hospital admission, underlying diseases, date of diagnosis of influenza A, length of the hospital stay, laboratory findings, department, ICU admission, use of corticosteroids, initial radiographic findings, and outcomes. Vaccination status was obtained by follow-up investigations with telephone calls.

According to previous reports [7, 9], the influenza A incubation period ranges from 1 to 7 days. To reflect pre-infection characteristics and avoid the influence from infection in the incubation period, we collected data of every hospital-acquired influenza A case for the date 7 days before the influenza A diagnosis, and used the same date for collecting the data of each matched control.

### Statistical analysis

Categorical variables were presented as frequencies and percentages. Continuous variables were presented as means and standard deviations (SDs) for data that followed a normal distribution, or as medians and interquartile ranges if the data distribution departed from normality. The characteristics of patients with hospital-acquired influenza A and their matched controls were compared. The t-test or Mann-Whitney U test was used for comparing continuous variables. The chi-squared test was used to compare categorical variables. Statistical significance was set at *P* < 0.05. All variables with *P* values < 0.15 in the univariate analysis, and sex, age and corticosteroid use were included in the conditional logistic regression analysis to identify the independent risk factors for hospital-acquired influenza A. In the regression analysis, the minimum sample size required was estimated empirically, equal to the number of independent variables multiplied by 20 [15]. The analysis was performed using SPSS version 20.0 statistical software (IBM Corp, Armonk, NY, USA).

## Results

### Epidemiological characteristics of influenza a in hospitalized patients

During the 2018–2019 influenza A season, we identified a total of 1336 hospitalized patients who had an ILI and were suspected to have influenza A (Fig. 1). The epidemiological characteristics of the suspected and confirmed influenza A in hospitalized patients are shown in Table 1. The positive rate of diagnosis among patients with suspected influenza A was significantly higher in January than in February, March and April (Supplemental Table S1). Suspected influenza A patients from the Nephrology and Geriatric Departments had higher positive rates of diagnosis (61.4 and 45.6%, respectively) than the overall rate (30.8%; *P* < 0.001 and *P* = 0.002, respectively; Supplemental Tables S2 and S3).

**Table 1:** Epidemiological characteristics of the suspected and the confirmed cases of influenza A

Variables	Suspected influenza A No.	RT-PCR positive No.	Positive rate %
Total patients	1336	412	30.8
Nephrology	70	43	61.4
Geriatric	103	47	45.6
Neurology	124	46	37.1
Hematology	86	28	32.6
Cardiac Surgery	114	35	30.7
Cardiology	118	36	30.5
Gastroenterology	124	33	26.6
Rest departments	597	144	24.1
Sex			
Male	764	232	30.4
Female	572	180	31.5
Influenza A season			
December 2018	14	5	35.7
January 2019	673	278	41.3
February 2019	308	83	26.9
March 2019	235	29	12.3
April 2019	106	17	16.0
Age, years			
> 65	336	111	33.0
> 18 ~ 65	901	271	30.1
> 12 ~ 18	34	9	26.5
> 6 ~ 12	12	4	33.3
≥ 6	53	17	32.1

**Fig. 2:**
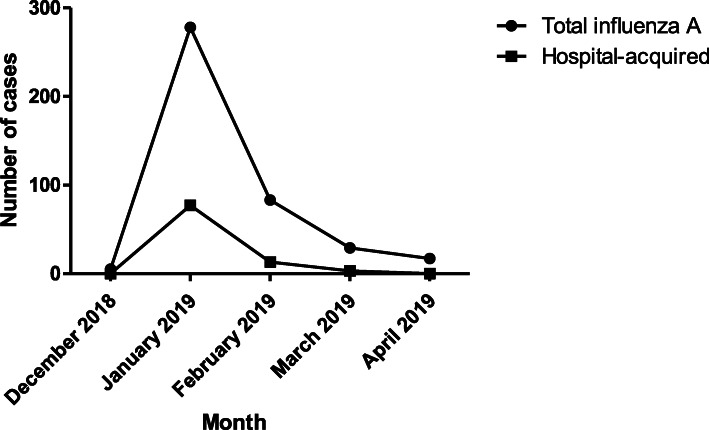
An epi curve of the total influenza A cases and the hospital-acquired cases

A total of 93 cases diagnosed 7 days or more after admission were classified as having hospital-acquired influenza A, and the temporal distribution characteristics are shown in Fig. 2. The epidemiological characteristics are shown in Table 2. Of these cases, 68.8% were confirmed 7 to 14 days after admission, and 31.2% were confirmed more than 14 days after admission. Twenty-two cases (23.7%) had a history of ICU admission during their hospitalization, including the nine that were confirmed after discharge from ICU and the thirteen that were diagnosed during hospitalization in ICU.

**Table 2:** Epidemiological characteristics of the 93 patients with hospital-acquired influenza A

Variables	Hospital-acquired influenza A No.	Distribution of the cases %
Total patients	93	100
Department		
Geriatric	15	16.1
Neurology	15	16.1
Hematology	13	14.0
cardiac surgery	12	13.0
Nephrology	6	6.5
Gastroenterology	6	6.5
Respiratory	5	5.4
Rest	21	22.6
ICU admission	22	23.7
Diagnosed time		
December 2018	0	0.0
January 2019	77	82.8
February 2019	13	14.0
March 2019	3	3.2
April 2019	0	0.0
Sex		
Male	50	53.8
Female	43	46.2
Age > 65 years	32	34.4

*ICU* Intensive care unit;

### Clinical characteristics of patients with hospital-acquired influenza a

Table 3 shows demographic data, underlying diseases, laboratory findings, and radiographic findings 7 days prior to the diagnosis of hospital-acquired influenza A. Approximately 90.3% of the infected patients had underlying chronic diseases. Hypertension, coronary heart disease, and cerebrovascular disease were the most common comorbidities, while 26.9% of patients were diagnosed pneumonia on admission. Compared with the controls, the cases had a higher prevalence of lymphocytopenia (51.6% vs 35.5%, *P* = 0.027), a lower median lymphocyte count (1070 vs 1300 cells/μL, *P* = 0.045), and a higher prevalence of anemia (55.9% vs 45.2%, *P* = 0.142), hypoalbuminemia (78.5% vs 57.0%, *P* = 0.002), pleural effusion (26.9% vs 9.7%, *P* = 0.002), and a higher frequency of corticosteroid use (50.5% vs 43.0%, *P* = 0.304) prior to the infection of influenza A (Table 3). Notably, patients with hospital-acquired influenza A had a longer hospital stay (median 18 vs 14 days, *P* = 0.002) and higher mortality rate (10.8% vs 2.2%, *P* = 0.017) (Table 3).

**Table 3:** The patient characteristics prior to the infection of hospital-acquired influenza A

Variables	Hospital-acquired influenza A No. (%)	Matched controls No. (%)	*P* value
Age, years, median (IQR)	58 (41.50–69.0)	59 (43.50–68.50)	0.971
Sex			
Male	50 (53.8)	49 (52.7)	0.883
History of smoking	24 (25.8)	20 (21.5)	0.490
Underlying disease			
Hypertension	39 (41.9)	34 (36. 6)	0.453
Diabetes	16 (17.2)	14 (15.1)	0.690
COPD	6 (6.5)	7 (7.5)	0.774
Coronary heart disease	20 (21.5)	22 (23.7)	0.726
Chronic renal failure	3 (3.2)	1 (1.1)	0.621
Malignancy^a^	6 (6.5)	8 (8.6)	0.578
Immunosuppression^b^	15 (16.1)	16 (17.2)	0.844
Hematologic disease	13 (14.0)	13 (14.0)	1.000
Cerebrovascular disease	19 (20.4)	15 (16.1)	0.448
Autoimmune disease	14 (15.1)	10 (10.8)	0.382
Pregnancy	1 (1.1)	1 (1.1)	1.000
Pneumonia on admission	25 (26.9)	19 (20.4)	0.301
Laboratory findings			
Leukocyte count, /mm^3^, median (IQR)	7000 (4700–9350)	6300 (4650–9700)	0.691
Leukocytopenia^c^	16 (17.2)	10 (10.8)	0.205
Neutrophilic granulocyte count, /mm^3^, median (IQR)	4650 (2780–6970)	3950 (2240–6410)	0.415
Neutrophilopenia^d^	16 (17.2)	11 (11.8)	0.298
Lymphocyte count, /mm^3^, median (IQR)	1070 (630–1660)	1300 (880–1820)	0.045
Lymphocytopenia^e^	48 (51.6)	33 (35.5)	0.027
Hemoglobin, g/L, median (IQR)	108 (87.5–133.5)	119 (97–139.5)	0.068
Anaemia^f^	52 (55.9)	42 (45.2)	0.142
Platelet count, /mm^3^, median (IQR)	180,000 (111500–256,500)	180,000 (141000–250,000)	0.351
Thrombocytopenia^g^	25 (26.9)	17 (18.3)	0.161
ALT, IU/L, median (IQR)	22 (14.5–35.5)	24 (16–37.5)	0.446
ALT > 50 IU/L	15 (16.1)	11 (11.8)	0.398
AST, IU/L, median (IQR)	22 (17–36.5)	22 (17.5–35)	0.601
AST > 40 IU/L	18 (19.4)	16 (17.2)	0.704
ALB, g/L, median (IQR)	35.6 (31.55–39.15)	38.5 (34.75–42.20)	0.001
Hypoalbuminemia^h^	73 (78.5)	53 (57.0)	0.002
TBIL, μmol/L, median (IQR)	12.4 (8.2–19.5)	13.7 (8.75–17.05)	0.691
TBIL > 20.5 μmol/L	18 (19.4)	17 (18.3)	0.851
DBIL, umol/L, median (IQR)	5.1 (3.2–8.55)	5.3 (3.1–7.75)	0.932
DBIL > 6.8 μmol/L	32 (34.4)	31 (33.3)	0.877
BUN, mmol/L, median (IQR)	5.62 (4.25–7.57)	5.68 (4.71–7.76)	0.622
BUN > 8 mmol/L	21 (22.6)	22 (23.7)	0.862
CRE, μmol /L, median (IQR)	61 (50.5–81.5)	60 (48.5–76.0)	0.489
CRE > 97 μmol /L	13 (14.0)	14 (15.1)	0.835
K^+^, mmol/L, mean (SD)	4.12 (0.62)	4.03 (0.49)	0.254
K^+^ < 3.5 mmol/L	14 (15.1)	12 (12.9)	0.672
Na^+^, mmol/L, mean (SD)	139.91 (4.81)	140.02 (4.44)	0.873
Na^+^ < 137 mmol/L	25 (26.9)	19 (20.4)	0.301
Ca^+^, mmol/L, mean (SD)	2.14 (0.19)	2.17 (0.17)	0.157
Ca^+^ < 2.11 mmol/L	37 (39.8)	30 (32.3)	0.285
Radiographic findings			
Pleural effusion^i^	25 (26.9)	9 (9.7)	0.002
Corticosteroid^j^	47 (50.5)	40 (43.0)	0.304
Corticosteroid, days, median, (IQR)	6 (3–7)	6 (3–8.5)	0.464
Influenza vaccine^k^	1/76 (1.3)	2/85 (2.4)	1.000
Length of hospital stay, days, median (IQR)	18 (12–27.5)	14 (11–20)	0.002
Mortality	10 (10.8)	2 (2.2)	0.017

Data are expressed as frequencies and percentages in parenthesis unless otherwise stated; *IQR* Interquartile range (25th to 75th percentile); *SD* Standard deviation

*COPD* Chronic obstructive pulmonary disease; *ALT* Alanine aminotransferase; *AST* Aspartate aminotransferase; *ALB* Albumin; *TBIL* Total bilirubin; *DBIL* Direct Bilirubin; *BUN* Blood urea nitrogen; *CRE* Creatinine; *K*^*+*^ Plasma potassium; *Na*^*+*^ Plasma sodium; *Ca*^*+*^ Serum calcium;

^a^ Malignancy: cancer or hematological malignancies;

^b^ Immunosuppression: chemotherapy or radiotherapy within 1 month before illness onset, using immunosuppressive therapy with a daily dose of ≥20 mg prednisolone (or its equivalent) for more than 15 continuous days before onset of the illness, and hematopoietic stem cells or solid organ transplant < 10 years

^c^ Leukocytopenia: leukocyte count < 3500/mm^3^

^d^ Neutrophilopenia: neutrophilic granulocyte count < 1800/mm^3^

^e^ Lymphocytopenia: lymphocyte count < 1100/mm^3^

^f^ Anemia: hemoglobin < 120 g/L for men and < 110 g/L for women

^g^ Thrombocytopenia: platelet count < 125 /mm^3^

^h^ Hypoalbuminemia: ALB < 40 g/L

^i^ Pleural effusion: on single or both sides found by radiographic

^j^ Corticosteroid: intravenous drip or atomizing inhalation

^k^ Influenza vaccine: vaccination coverage was 1 in 76 and 2 in 85, respectively, due to death and loss of follow-up

The median age of the hospital-acquired influenza A patients with fatal outcome was 90.5 years (range, from 39 to 94 years). Six cases with poor baseline physical conditions on admission were from the Geriatric Department, and were aged from 90 to 94 years. Two patients, aged 89 and 68 years, were from the Neurology Department, and both had pre-existing cerebrovascular disease and were bedridden, with protracted and intractable pneumonia on admission. One patient, aged 39 years, was from the Cardiac Surgery Department, and had rheumatic heart disease with atrial fibrillation, as well as severe myocardial injury from a mitral valve replacement. One patient, aged 56 years, was from the Hematology Department, and was immunosuppressed with diffuse large B-cell lymphoma.

### Risk factors for hospital-acquired influenza a

Univariate analysis (Table 3) showed that lymphocytopenia, hypoalbuminemia, and pleural effusion can be associated with hospital-acquired influenza A. These three risk factors and anemia (*P* = 0.142 < 0.15), in addition to sex, age, and corticosteroid use, were included in the conditional logistic regression analysis. Based on the empirical estimation, the minimum total sample size required was 140; therefore, the study sample size (*N* = 186 for cases and controls combined) was adequate. The logistic regression analysis revealed that lymphocytopenia (OR: 3.11, 95% CI: 1.24–7.80, *P* = 0.016), hypoalbuminemia (OR: 2.24, 95% CI: 1.10–4.57, *P* = 0.027) and pleural effusion (OR: 3.09, 95% CI: 1.26–7.58, *P* = 0.014) were independent risk factors for hospital-acquired influenza A (Table 4).

**Table 4:** Independent risk factors for hospital-acquired influenza A

Variables	OR	95%CI	*P* value
Lymphocyte count			
≥ 1100/mm^3^	1 (reference)		
Lymphocytopenia	3.11	1.24–7.80	0.016
Albumin			
≥ 40 g/L	1 (reference)		
Hypoalbuminemia	2.24	1.10–4.57	0.027
Radiographic findings			
No pleural effusion	1 (reference)		
Pleural effusion	3.09	1.26–7.58	0.014

Lymphocytopenia: lymphocyte count < 1100/mm^3^; Hypoalbuminemia: albumin < 40 g/L; Pleural effusion: on single or both sides found by radiographic

## Discussion

To the best of our knowledge, this is the first retrospective matched case-control study of risk factors for hospital-acquired influenza A that included all the different departments of a hospital over a single full influenza A season. This is the first study to identify lymphocytopenia, hypoalbuminemia, and pleural effusion as independent risk factors for hospital-acquired influenza A.

In China, according to the overview of epidemic situation of statutory infectious diseases provided by the National Health Commission, the number of influenza (A, B, and C) cases from January 2019 to April 2019 was 1.575 million which is considerably more than the 0.768 million cases reported for the 2018 year [16, 17]. Influenza weekly statistics released by the Chinese National Influenza Center showed that in the first, fifth, and ninth week of 2019, influenza A virus was the main pathogen, accounting for 99.5, 98.1, and 89.9% of influenza cases in the Northern Provinces of China, respectively [18]. In our study, hospital-acquired influenza A cases occurred mainly in January and February 2019. Furthermore, according to the influenza weekly, H1N1 subtype accounted for 92.7 and 86.9% of influenza A in the first and fifth week of 2019 in Northern China, respectively [18].

Our study has two main strengths. First, with a strict case definition and a rigorous paired design, we consider that our comparative analysis is more reliable than those of previous studies [7, 12]. Second, hospital-acquired influenza A cases were selected from all the different departments of the hospital in a single influenza A season, indicating that hospital-acquired infection was caused by identical or similar influenza A virus strains, which ensured the comparability and homogeneity of clinical data.

Approximately 23% of the influenza cases diagnosed in our study were classified as hospital-acquired. This large proportion of cases may be because of the large increase in the number of influenza cases nationwide in China in early 2019 compared with previous years. Studies conducted in other countries have had similar findings. In a tertiary care hospital in France, during the 2016–2017 influenza A season, 25% of hospitalized patients with influenza A were considered to be hospital-acquired [12]. A German university hospital reported 24% hospital-acquired infection cases in the 2012–2013 influenza season and 20% in the 2013–2014 season [5]. However, a lower proportion of hospital-acquired cases have been observed in other studies. In the UK, during the 2009 H1N1 pandemic, 2% of hospitalized cases with influenza A were considered hospital-acquired [19]. In an epidemiological study based on the data of six influenza seasons from 2010 to 2011 to 2015–2016 in Spain, of the hospitalized patients with confirmed severe influenza, 5.6% were classified as hospital-acquired [20].

This variability in the prevalence of hospital-acquired influenza A can be attributed to variations in study design and differences between regions and strains of virus. Currently, there is no consensus with respect to the criteria required for declaring an influenza outbreak in a hospital. According to some studies, a hospital-acquired influenza outbreak is defined by an increase in cases of hospital-acquired influenza in a short time and limited space [21, 22].

The suspected patients from the Nephrology and Geriatric Departments had a higher positive rate of diagnosis than the average of the hospital. Most patients in the Nephrology Department had chronic kidney diseases, leading to a range of immune system defects [23] such as decreased chemotaxis and phagocytosis of monocyte/macrophage, B-cell lymphopenia, and depressed CD4^+^ and CD8^+^ T cell responses [24]. Therefore, patients in the Nephrology Department were more vulnerable to the morbidity and mortality associated with influenza infections [25]. Corticosteroids are commonly used drugs in the Nephrology Department and recent research has shown that corticosteroid use can enhance the replication of respiratory viruses [26]. However, corticosteroid use was not a significant risk factor for hospital-acquired influenza A in our study, probably because we matched cases with controls from the same department, and thus the cases and controls were treated with similar medications. It has been reported that age > 65 years was a risk factor for influenza [27]. Thus, suspected patients in the Geriatric Department in a poor basic physical condition can be more likely to test positive for influenza A. During annual seasonal influenza A epidemics, it is recommended that the Nephrology and Geriatric Departments pay more attention to disease prevention and diagnosis.

Due to death and loss of follow-up, we were unable to ascertain the vaccination status of a few patients. However, the vaccination uptake among both the hospital-acquired influenza A cases and the controls was only 1.3 and 2.4%, respectively, a difference that was not statistically significant. The coverage of influenza vaccine in the overall Chinese population was reported to be 0.8–2.2% during 2004 to 2014 [28]. Furthermore, in 2017, of the 379 adults aged ≥60 years who were interviewed in a developed city in eastern China, only 0.8% reported receiving the influenza vaccine in the previous year [29]. Currently, self-paid vaccination, unawareness of the need, and few recommendations from medical staff contribute to the low vaccine coverage. By contrast, the vaccination coverage among older people in England, France and Germany reached 73, 49 and 37% respectively during the 2014–2015 season [30].

There are several reasons why hospital-acquired viral infections are less likely to be reported than hospital-acquired bacterial infections, including historical attention to bacterial infection [31], difficulties in diagnosis of viral infections, and limited availability of antiviral drugs [11]. Droplet precautions with single room isolation, as an important infection control procedure, are required for all suspected or confirmed cases. This consumes a vast amount of medical resources, creating challenges in the prevention and control of hospital-acquired influenza A. Notably, our study showed that hospital-acquired influenza A carried a considerable risk of death and prolonged hospital stay compared to the control group.

Our findings indicated that the Geriatric and Neurology Department had the highest number of hospital-acquired influenza A cases, followed by the Hematology and Cardiac Surgery Departments. During the influenza season, patients who underwent cardiac surgery were more likely to develop acute respiratory distress syndrome (ARDS) [32]. In this study, all patients from the Cardiac Surgery Department had undergone surgery before they acquired influenza A, however we were unable to determine whether cardiac surgery increases the risk of influenza A. This question requires further investigation.

Lymphopenia is common among patients with influenza A [33] and is associated with poor outcomes [34]. Influenza viral replication is initially controlled by innate immunity and thereafter adaptive immune responses (T cells and antibody-producing B cells), lead to viral clearance and host recovery [35]. This may explain the frequent outbreaks of influenza A in Hematology Departments that have occurred over many years [4, 36, 37].

Hypoalbuminemia is the result of the combined effect of inflammation and inadequate protein and caloric intake in patients with chronic disease such as chronic renal failure [38]. Hypoalbuminemia is frequently observed in hospitalized patients, therefore early detection of vulnerable individuals is essential for implementation of infection control. While we do not recommend albumin supplementation for patients with hypoalbuminemia for the prevention of influenza A, we recommend that measures such as droplet precautions with single room isolation be mandatory.

Pleural effusion, a radiographic finding, can be caused by hypoalbuminemia, pleural infections, heart and kidney failure, pulmonary embolism or malignancy. We did not quantify the amount of effusion or differentiate between single-sided and bilateral effusions, but used it as a qualitative diagnosis. Two studies from Taiwan reported that in pediatric influenza patients, radiographically-confirmed pleural effusion on admission was significantly associated with a severe infection that required intensive care [39, 40]. Having a pleural effusion might make patients more vulnerable to the influenza A virus through an unknown mechanism.

There are some limitations to our study. We were not able to meet the requirement for the design that every hospital-acquired influenza A patient should be matched with a ideal control case, who had been hospitalized for 7 days or more in the same room on the date when the paired case acquired influenza. To ensure a sufficient sample size, we were only able to match the same department, but not the same room. This may have reduced the comparability of the two groups. However, two matched patients from the same department were exposed to the same aerosol environment, which played an important role in the spread of the infection. In addition, a relatively stringent definition of hospital-acquired influenza A was adopted, therefore some cases may have been missed and the true risk of acquiring influenza A in the hospital, underestimated. Finally, the clinical data from patients hospitalized for more than 7 days because of severe primary diseases may have exaggerated the impact of hospital-acquired infections, thus the clinical characteristics of hospital-acquired influenza A may be overstated.

## Conclusions

This study shows that hospital-acquired influenza A can extend hospital stay and is associated with a high mortality rate; thus, its prevention requires more attention. Precautions need to be taken to protect hospitalized patients who present with lymphocytopenia, hypoalbuminemia, or pleural effusion, due to their increased risk of hospital-acquired influenza A. Further prospective studies with large sample sizes need to be carried out to confirm our findings.

## Supplementary Information

**Additional file 1:** **Supplemental Table S1.** Pairwise comparison of the positive rates of diagnosis among suspected patients in the five months. **Supplemental Table S2.** Comparison of the positive rates of diagnosis among suspected patients from the Nephrology Department and the whole hospital. **Supplemental Table S3.** Comparison of the positive rates of diagnosis among suspected patients from the Geriatric Department and the whole hopital.

## Data Availability

The datasets used and/or analyzed during this study are available from the corresponding author on reasonable request.

## Abbreviations

ALB: Albumin
ALT: Alanine aminotransferase
AST: Aspartate aminotransferase
ARDS: Acute respiratory distress syndrome
BUN: Blood urea nitrogen
COPD: Chronic obstructive pulmonary disease
CRE: Creatinine
CRF: Case report form
DBIL: Direct bilirubin
ICU: Intensive care unit
ILI: Influenza-like illness
IQR: Interquartile range
RT-PCR: Real-time reverse transcriptase polymerase chain reaction
SD: Standard deviation
TBIL: Total bilirubin
