# Supplementary material for: Risk factors for hospital-acquired influenza A and patient characteristics: a matched case-control study
Source: BMC Infect Dis. 2020 Nov 19;20:863. doi: 10.1186/s12879-020-05580-9 (PMC7675395; doi:10.1186/s12879-020-05580-9)
Supplement: Supplementary file 1 — Additional file 1: Supplemental Table S1. Pairwise comparison of the positive rates of diagnosis among suspected patients in the five months. Supplemental Table S2. Comparison of the positive rates of diagnosis among suspected patients from the Nephrology Department and the whole hospital. Supplemental Table S3. Comparison of the positive rates of diagnosis among suspected patients from the Geriatric Department and the whole hopital. [file 12879_2020_5580_MOESM1_ESM.docx]

**Supplementary Materials**

**Supplemental Table S1** Pairwise comparison of the positive rates of diagnosis among suspected patients in the five months

| Between-group comparisons | χ² | P value |
| --- | --- | --- |
| January 2019 vs February 2019 | 18.733 | ＜0.001 |
| January 2019 vs March 2019 | 65.308 | ＜0.001 |
| January 2019 vs April 2019 | 24.854 | ＜0.001 |
| January 2019 vs December 2018 | 0.177 | 0.674 |
| February 2019 vs March 2019 | 17.373 | ＜0.001 |
| February 2019 vs April 2019 | 5.124 | 0.024 |
| February 2019 vs December 2018 |  | 0.541 |
| March 2019 vs April 2019 | 0.856 | 0.355 |
| March 2019 vs December 2018 |  | 0.028 |
| April 2019 vs December 2018 |  | 0.132 |

December 2018 vs February 2019, March 2019 and April 2019 were compared by Fisher's exact test. Statistical significance was set at P < 0.005.

**Supplemental Table S2** Comparison of the positive rates of diagnosis among suspected patients from the Nephrology Department and the whole hospital

|  | Suspected influenza A No. | | RT-PCR positive  No. (%) |  |
| --- | --- | --- | --- | --- |
| Nephrology | 70 | | 43（61.4） |  |
| All departments | 1336 | | 412（30.8） |  |
| χ² | | 28.43 | | |
| P value | | ＜0.001 | | |

**Supplemental Table S3** Comparison of the positive rates of diagnosis among suspected patients from the Geriatric Department and the whole hopital.

|  | Suspected influenza A No. | | RT-PCR positive  No. (%) |  |
| --- | --- | --- | --- | --- |
| Geriatric | 103 | | 47（45.6） |  |
| All departments | 1336 | | 412（30.8） |  |
| χ² | | 9.633 | | |
| P value | | 0.002 | | |
